# Gurvits Syndrome: Black Esophagus in the Postoperative Setting

**DOI:** 10.7759/cureus.21240

**Published:** 2022-01-14

**Authors:** Mahrukh Ali, Noman Khan, Asma Yaseen, Aimun Raees

**Affiliations:** 1 Gastroenterology and Hepatology, Aga Khan University Hospital, Karachi, PAK

**Keywords:** postoperative, esophageal necrosis, setting, black esophagus, gurvits syndrome

## Abstract

The “black esophagus” or “acute esophageal necrosis” is a very rare condition of the esophagus that is believed to have a multifactorial etiology and usually involves the distal esophagus. We present a case of a 66-year-old gentleman who presented with a history of retching followed by one episode of hematemesis two days after his left inguinal hernioplasty. His esophagogastroduodenoscopy showed diffuse ulceration and erosions throughout the esophagus, necrosis of the distal esophageal mucosa till the gastroesophageal junction, and a healed ulcer in the first part of the duodenum. He was managed with supportive care and discharged home in a stable condition.

## Introduction

The “black esophagus” or “acute esophageal necrosis” is a very rare condition of the esophagus that is believed to have a multifactorial etiology and usually involves the distal esophagus [1]. The most common presentation is upper gastrointestinal bleed. However, these patients may present with abdominal pain, dysphagia, and esophageal perforation in some severe cases [2,3]. It has been hypothesized that gastroesophageal reflux of acidic contents causing recurrent injury to the esophageal mucosa and hypoperfusion of the esophagus in conditions such as acute heart failure and septic shock are responsible for the development of esophageal necrosis [4,5]. In an autopsy series, the prevalence of esophageal necrosis was found to be 0.2% [6]. The microvascular involvement in patients with diabetes mellitus may also predispose them to the development of esophageal necrosis [7].

## Case presentation

**Figure 1:**
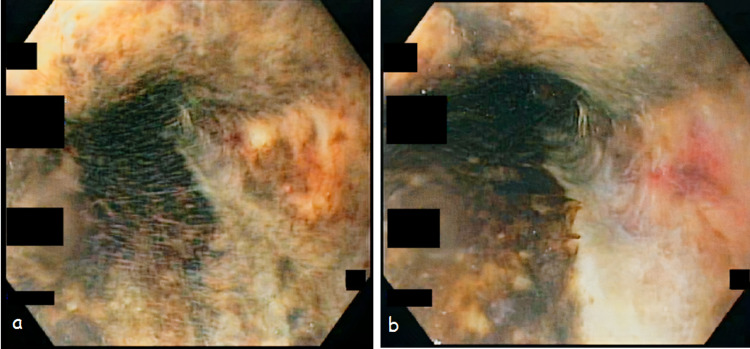
A, B: Esophagogastroduodenoscopy images showing blackish discolored necrotic mucosa of the distal esophagus.

A 66-year-old male presented with a history of persistent retching followed by one episode of hematemesis two days after his left inguinal hernioplasty. He was a known case of hypertension, gastroesophageal reflux disease, and ischemic heart disease and was on dual antiplatelet therapy with a baseline ejection fraction of 45%. On arrival in the emergency room, he had tachycardia with a heart rate of 115 beats per minute but was maintaining his blood pressure and oxygen saturation. He was resuscitated with intravenous normal saline infusion, following which intravenous omeprazole infusion was started. His hemoglobin levels dropped from the baseline of 15.2 g/dL (normal range: 12.3-16.6 g/dL) to 11.3 g/dL. However, blood transfusion was not needed throughout the hospital stay as he maintained his hemoglobin and remained hemodynamically stable. After the initial resuscitation, we performed an esophagogastroduodenoscopy (EGD) that showed diffuse ulceration and erosions throughout the esophageal length, necrosis of the distal esophageal mucosa till the gastroesophageal junction (Figure 1), and a healed ulcer in the first part of the duodenum. His hospital course was uneventful and he was discharged after three days. He was discharged on proton pump inhibitors and empirical anti-*Helicobacter pylori* eradication therapy with an early follow-up and a plan of re-look EGD after a few weeks. His antiplatelet therapy was stopped until the follow-up.

## Discussion

The black esophagus is a rare clinical condition most commonly involving the distal part of the esophagus [1,4]. It is mostly seen in the elderly usually in the sixth decade with a male predominance of 2.3:1 [8]. Endoscopic appearance shows a diffuse blackish discoloration of the distal esophagus that affects it circumferentially [9]. Although the etiology of this condition remains unclear, it is likely multifactorial in origin. A combination of two factors, one that can possibly lead to mucosal injury, that is, reflux of gastric contents, and another that decreases the blood supply of the esophagus such as shock, is thought to be involved in the pathogenesis of this disease [4,5,10]. This “two-hit” model of ischemia and acid reflux injury has been proposed in the literature as a possible explanation of esophageal necrosis [11].

Multiple comorbidities can precipitate this condition. Diabetes mellitus is one of the common accentuating factors, and hyperglycemia is found in 90% of the patients presenting with esophageal necrosis [12]. Malnutrition has also been suggested to be one of the factors involved in accelerating the disease process [13]. Other risk factors mentioned in previous reports include gastric outlet obstruction, gastric volvulus, shock, aortic dissection, diabetic ketoacidosis, alcohol abuse, etc. The treatment is usually supportive care by keeping the patient nil per oral, intravenous fluid resuscitation, broad-spectrum antibiotics, acid-suppressing agents, oral sucralfate suspension, and avoidance of factors that can precipitate the illness such as hyperglycemia. Short-term total parenteral nutrition can be given to malnourished individuals.

Although perforation is rare, it is the most fatal acute complication of esophageal necrosis. If it occurs, esophagectomy is the surgical management [14]. Stricture formation is also seen in the chronic phase of the disease in some patients. Patients who present with a critical illness have a high rate of mortality [1,2]. The mortality of esophageal necrosis ranges from 30% to 50% depending upon the etiology [4].

## Conclusions

Acute esophageal necrosis is a rare condition that usually involves the distal esophagus. Its etiology is not clear yet but is suggested to be multifactorial. A “two-hit” model of mucosal injury due to reflux of gastric contents and decrease in esophageal blood supply has been proposed. Patients presenting with a critical condition have high mortality. The treatment is mostly cause-directed and supportive care.
